# Quantitative ^129^Xe MRI detects early impairment of gas-exchange in a rat model of pulmonary hypertension

**DOI:** 10.1038/s41598-020-64361-1

**Published:** 2020-04-30

**Authors:** Rohan S. Virgincar, John C. Nouls, Ziyi Wang, Simone Degan, Yi Qi, Xinyu Xiong, Sudarshan Rajagopal, Bastiaan Driehuys

**Affiliations:** 1 Department of Biomedical Engineering, Duke University, Durham, NC USA; 2 Department of Radiology, Duke University Medical Center, Durham, NC USA; 3 Department of Medicine, Duke University Medical Center, Durham, NC USA

**Keywords:** Diagnostic markers, Hypertension, Magnetic resonance imaging, Preclinical research

## Abstract

Hyperpolarized ^129^Xe magnetic resonance imaging (MRI) is capable of regional mapping of pulmonary gas-exchange and has found application in a wide range of pulmonary disorders in humans and animal model analogs. This study is the first application of ^129^Xe MRI to the monocrotaline rat model of pulmonary hypertension. Such models of preclinical pulmonary hypertension, a disease of the pulmonary vasculature that results in right heart failure and death, are usually assessed with invasive procedures such as right heart catheterization and histopathology. The work here adapted from protocols from clinical ^129^Xe MRI to enable preclinical imaging of rat models of pulmonary hypertension on a Bruker 7 T scanner. ^129^Xe spectroscopy and gas-exchange imaging showed reduced ^129^Xe uptake by red blood cells early in the progression of the disease, and at a later time point was accompanied by increased uptake by barrier tissues, edema, and ventilation defects—all of which are salient characteristics of the monocrotaline model. Imaging results were validated by H&E histology, which showed evidence of remodeling of arterioles. This proof-of-concept study has demonstrated that hyperpolarized ^129^Xe MRI has strong potential to be used to non-invasively monitor the progression of pulmonary hypertension in preclinical models and potentially to also assess response to therapy.

## Introduction

Pulmonary arterial hypertension (PAH) is a rare and particularly severe form of pulmonary hypertension (WHO Group 1), characterized histopathologically by endothelial cell apoptosis, smooth-muscle proliferation, and obliteration of pulmonary arterioles. These pathological changes result in a progressive increase in pulmonary arterial pressure and pulmonary vascular resistance that eventually leads to right heart failure and death^1–5^. Despite the development of new pharmacotherapies, the treatment of PAH is limited, and morbidity and mortality associated with it is substantial, with a median survival of 7 years with current therapies^6,7^.

In order to characterize the pathogenesis of PAH, as well as to test novel pharmacologic interventions, a number of rodent models have been routinely employed. The most well-established and widely-used preclinical models in the rat are the Sugen/hypoxia (SuHx) and monocrotaline (MCT) based models^8^. While these models recapitulate some of the pathology of PAH—with vascular remodeling, an elevation in pulmonary arterial pressures, and right ventricular hypertrophy—they also have additional effects on lung airways and parenchyma, and therefore are best considered models of pulmonary hypertension (PH). Assessing these models non-invasively and longitudinally remains a significant challenge. The major non-invasive tools currently available are echocardiography and cardiac magnetic resonance imaging (MRI) to assess right ventricular function^9^. However, these technologies only provide a surrogate measure of pulmonary artery pressure, and the associated views of the right ventricle can be difficult to obtain and quantify outside of expert hands. To this end, studies of PH models must typically also include terminal procedures such as right-heart catheterization for assessing hemodynamics, and harvesting of tissues for histopathologic assessment^9^.

Beyond the need to sacrifice the animals, these methods have further shortcomings. For example, while right-heart catheterization enables direct measurement of pulmonary arterial pressure and hemodynamics, it requires anesthesia and invasive procedures that can alter normal heart function. Moreover, hemodynamics and pressures are a surrogate for the underlying arteriopathy of interest. Such arteriopathy can be directly visualized by histopathology at the sites of injury by confirming smooth muscle proliferation and obliteration of arterioles. However, it is difficult to assess the disease burden throughout the entire lungs and, if the disease is heterogeneous, there is a risk for sampling error. Moreover, unlike humans, rodent models often show discrepancies between hemodynamic measurements in the heart and histopathology^9^. Thus, existing methods for monitoring the progression of PH and testing novel therapies both require significant expertise, and still suffer substantial limitations.

These challenges can potentially be addressed by 3D isotropic non-invasive imaging, which can be used to directly detect vascular lesions at the site of injury in the lungs. However, conventional methods like ^1^H MRI and computed tomography are not sensitive enough to detect early vascular injury occurring on a histological scale. To this end, hyperpolarized (HP) ^129^Xe MRI has emerged as a powerful technique for high-resolution 3D imaging of lung structure and function. The ability to image ^129^Xe in pulmonary airspaces, its uptake in interstitial barrier tissues and its transfer to red blood cells (RBCs) enables direct probing of gas-exchange at the pulmonary-capillary level. To date such comprehensive 3D imaging of the ^129^Xe distribution in these three compartments has proven highly sensitive to ventilation, diffusion, and perfusion limitation, associated with a wide range of pulmonary disorders such as asthma, chronic obstructive pulmonary disease, idiopathic pulmonary fibrosis (IPF), and radiation induced lung injury^10–12^. However, the application of ^129^Xe MRI to PH has thus far been limited only to a single clinical case report wherein ^129^Xe transfer to RBCs was found to be severely diminished in two patients ultimately found to have pulmonary vascular disease^13^. This promising result suggests that ^129^Xe MRI, in the form of RBC-transfer imaging, exhibits sensitivity to altered pulmonary capillary hemodynamics that are a hallmark in PAH. However, such imaging and spectroscopic signatures of PAH remain to be fully characterized, not only in patients, but also in well-established animal models where imaging can be validated against ground truth histology.

In this study, we sought—for the first time—to apply 3D quantitative ^129^Xe gas-exchange MRI and spectroscopy combined with anatomical ^1^H MRI to a preclinical model of PH. For the study described here, the MCT model was selected owing to its reproducibility, and its ability to generate vascular lesions similar to early stage lesions in human PAH^14^. Such pulmonary vascular changes are typically noted 2 weeks after injection, and occur before the development of mild-to-moderate PH at 3 weeks that then progresses to severe PH at 4 weeks^15^. Notably, these features are preceded by ventilatory dysfunction, associated with increased alveolar wall thickness, which occur only 1 week after injection^16^. We hypothesized that ^129^Xe MRI would be sensitive to regionally impaired ^129^Xe transfer to RBCs, as well as to potential additional structural and functional defects that could be introduced by pneumotoxicity of MCT. Moreover, we hypothesized that such lesions would be detectable early in the disease progression, prior to the development of frank PH. To test these hypotheses, we employed a cross-sectional study design, in which groups of rats injected with MCT, underwent imaging at two early time points in the disease (1 week and 2 weeks post-injection, labeled MCT-wk1 and MCT-wk2), and were then sacrificed for histological validation of disease. The inclusion of multiple time points was intended to provide preliminary insight into the sensitivity of ^129^Xe MRI metrics to the early stages of disease in MCT PH.

## Results

### Histological validation of PAH

**Figure 1:**
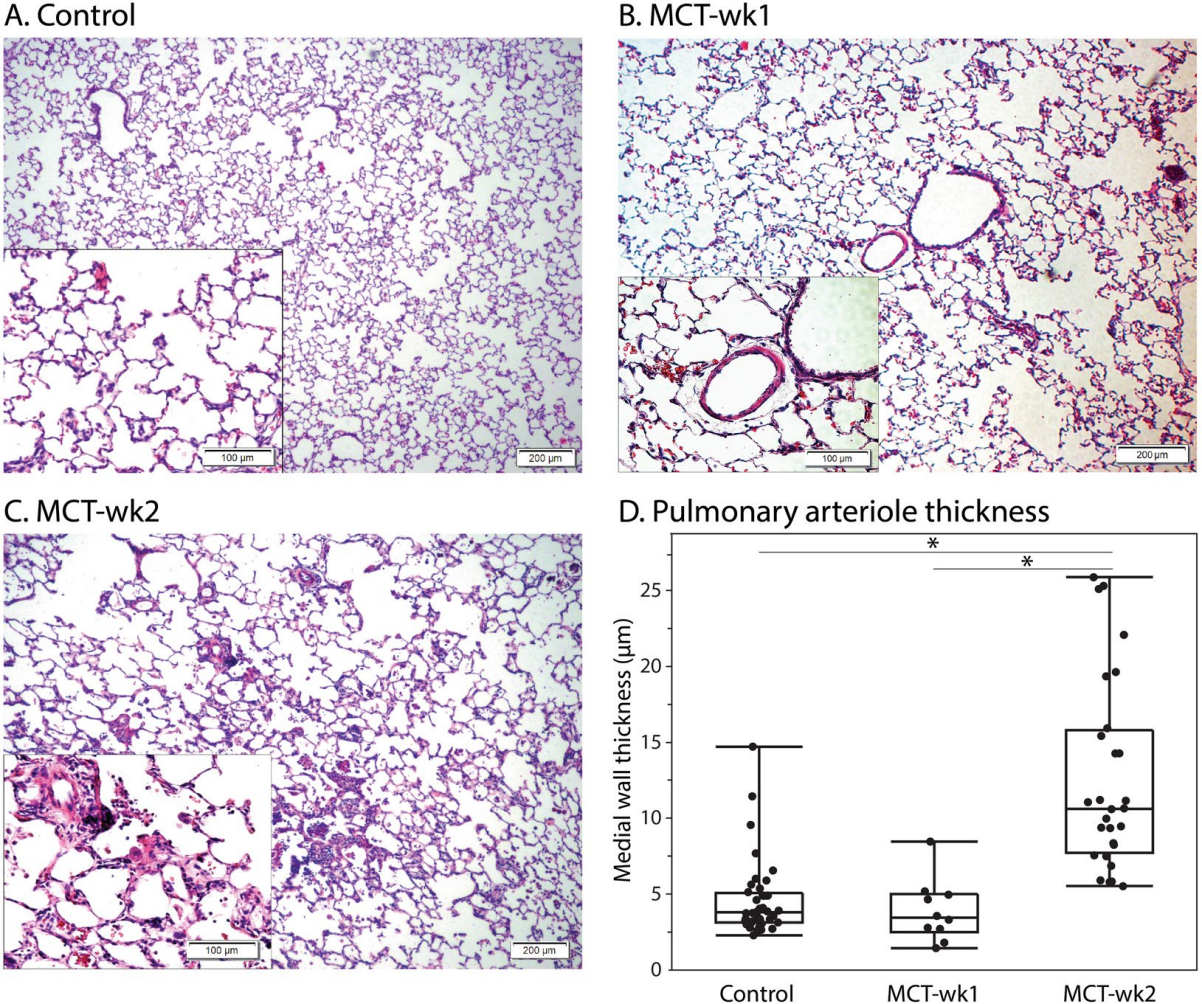
H&E stained histology slides obtained from a representative healthy control (**A**), and PH models (**B,C**). The disease models showed mild thickening of the endothelial layer, and moderate smooth muscle proliferation increasing from week-1 to week-2. (**D**) Development of medial wall thickness in the pulmonary arterioles, showing a significant increase at week-2 (P < 0.0001). Whiskers show minimum and maximum values.

Figure 1 shows H&E stained histology from control, MCT-wk1, and MCT-wk2 rats. The PH rats exhibited mild-to-moderate remodeling of the small arterioles (vessels that are 50–100 microns in diameter) compared to untreated animals. This was accompanied by mild thickening of the endothelial layer, and gradually increasing smooth muscle proliferation from week-1 to week-2. Notably, medial thickness was only significantly increased at week-2 (P < 0.0001), with no significant difference between control and week-1 rats (Fig. 1D, Table 1), consistent with the time-course of MCT effects^16^. Inflammation of the alveolar septum was also paired with an infiltration of macrophages and lymphocytes.

**Table 1:** Quantitative histology, ^129^Xe spectroscopy and MRI metrics for healthy and PH rats.

Group	HISTOLOGY	^129^Xe SPECTROSCOPY	^129^Xe MRI		
	Medial Wall Thickness (µm)	RBC:barrier	VDP	Barrier_high_%	RBC_defect_%
Control (N = 8)	4.6 ± 2.5	0.47 ± 0.03	2.5 ± 0.9	3.5 ± 1.9	11.8 ± 3.6
PH (N = 9)	10.3 ± 6.7	0.40 ± 0.06	3.3 ± 2.6	7.9 ± 6.3	17.1 ± 5.3
MCT-wk1 (N = 4)	3.9 ± 2.0	0.41 ± 0.04	1.5 ± 0.5	7.3 ± 8.1	17.6 ± 6.3
MCT-wk2 (N = 5)	12.6 ± 6.3	0.40 ± 0.08	4.7 ± 2.7	8.3 ± 5.4	16.7 ± 5.0

All values are reported as mean ± one standard deviation.

### Anatomical ^1^H MRI

**Figure 2:**
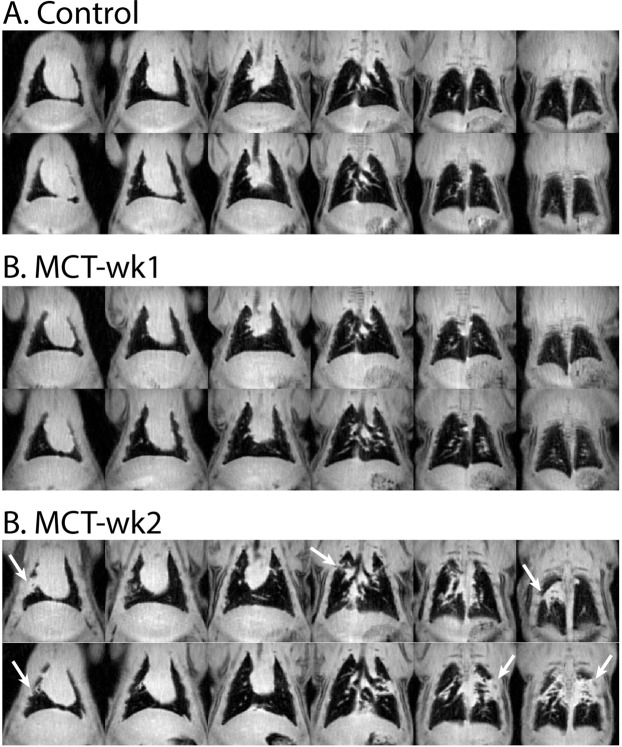
^1^H MRI (4-slice averages) in representative controls and PH models. MCT-wk2 rats showed extensive edema mostly in posterior and right anterior lung regions.

Figure 2 shows representative ^1^H MR images from control, MCT-wk1, and MCT-wk2 rats. The control and week-1 animal showed a clear, featureless thoracic cavity. At week-2, edema was observed in 4/5 animals and presented as large masses in the posterior lung (white arrows), and traces in the right-anterior lung.

### Hyperpolarized ^129^Xe spectroscopy

**Figure 3:**
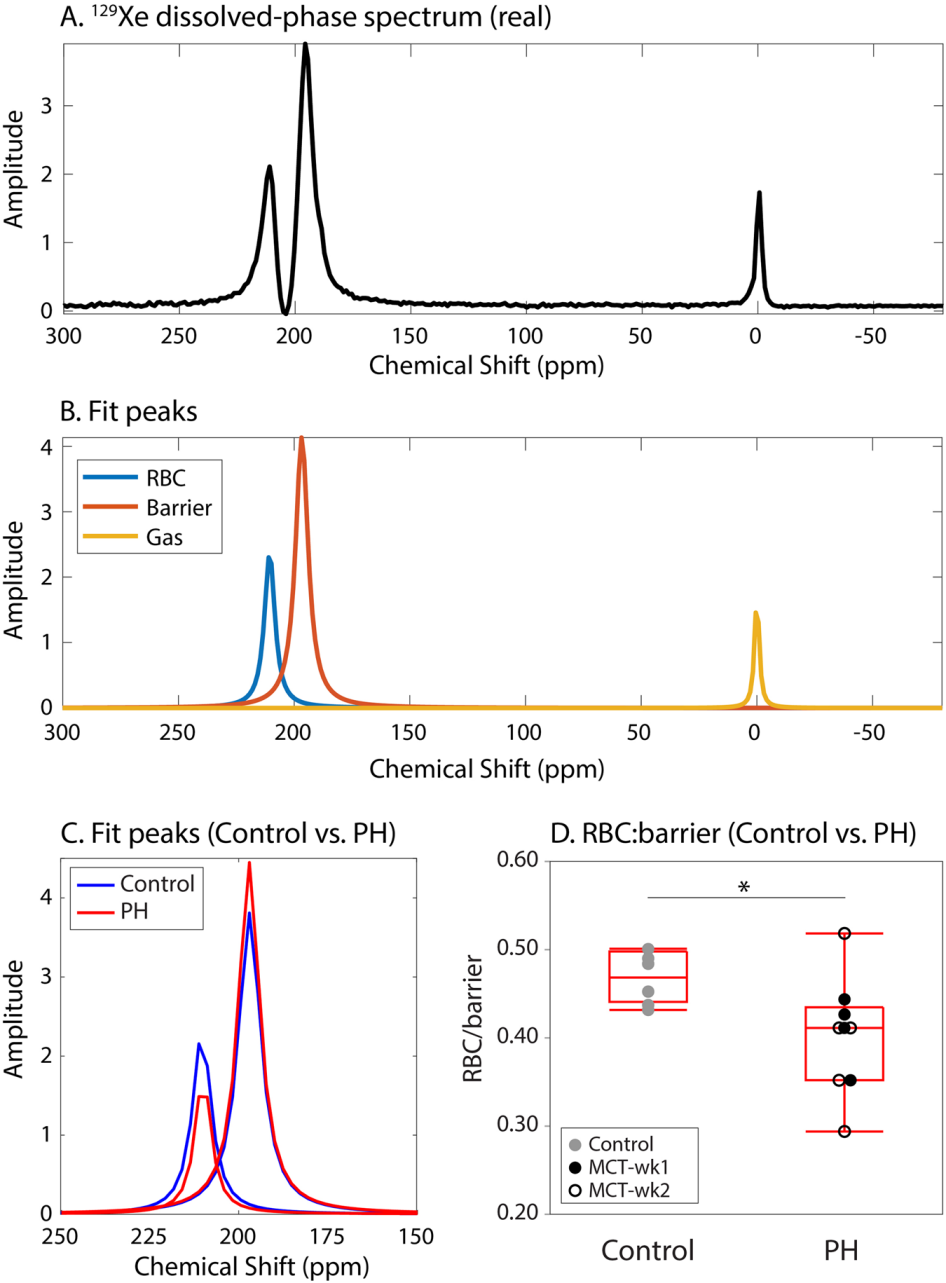
The ^129^Xe dissolved-phase spectrum (**A**) was fit to extract the gas-phase, barrier, and RBC resonances (**B**). In the PH group, the ratio of ^129^Xe signal in RBCs vs. barrier tissues (RBC:barrier) was found to be significantly reduced relative to the control group (**C,D**) (P = 0.014).

Figure 3A shows a representative dissolved-phase ^129^Xe spectrum in a healthy rat, which was decomposed into its gas-phase, barrier, and RBC resonances (Fig. 3B). Using the gas-phase resonance as the reference frequency, the RBC and barrier resonances had chemical shifts of 210.5 ± 0.1 ppm and 196.9 ± 0.2 ppm in the control group, and 210.7 ± 0.2 and 196.9 ± 0.2 ppm in the PH group.

Figure 3C demonstrates the ratio of signal from the barrier and RBC resonances from a PH animal (red) overlaid onto a control (blue). The PH animal shows diminished ^129^Xe signal from RBCs relative to barrier tissue, as quantified by the ratio of the amplitudes of the RBC and barrier resonances (RBC:barrier). The average RBC:barrier in the control group was 0.47 ± 0.03, whereas that in the PH group was significantly lower at 0.40 ± 0.06 (P = 0.014). RBC:barrier was observed to significantly reduce 1 week post MCT-injection (0.41 ± 0.04, P = 0.022) (Fig. 3D; Table 1). RBC:barrier further reduced at week-2 (0.40 ± 0.08), but this change was not statistically significant because of the large variability in the MCT-wk2 group. This variability is primarily driven by an outlier with RBC:barrier = 0.52, which showed ventilation impairment but no significant RBC defects relative to controls.

### Hyperpolarized ^129^Xe ventilation and gas-exchange mapping

**Figure 4:**
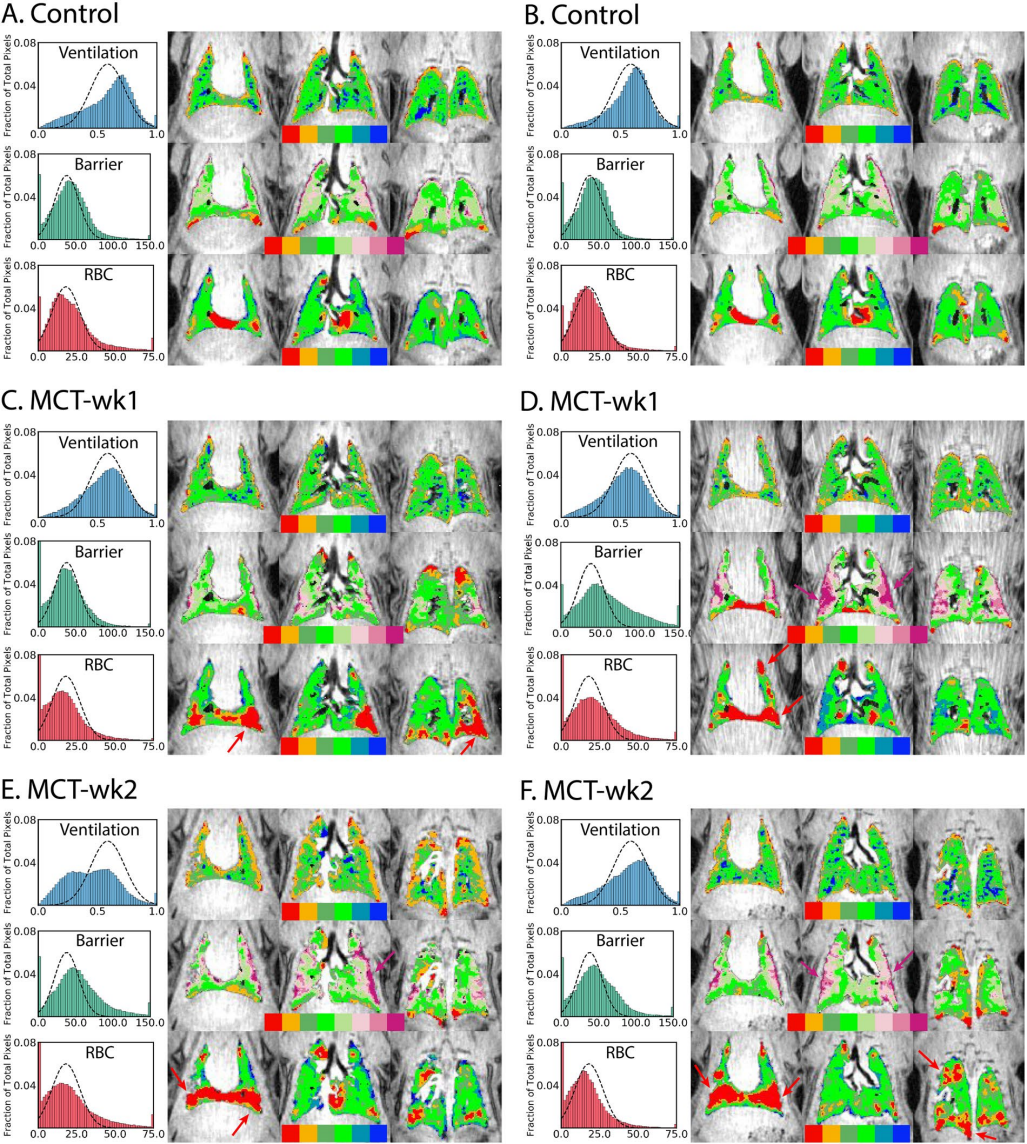
Representative ^129^Xe ventilation, barrier:gas, and RBC:gas maps with histograms in healthy and PH rats. The dotted line in the histogram represents the reference distribution derived from healthy animals. The maps from the control group show homogeneous ventilation, barrier-uptake, and RBC-transfer signal with a few baseline defects in barrier and RBC signal, mostly confined to the base of the lung. Gas-exchange maps in PH models show reduced ^129^Xe uptake in RBCs at week-1 and week-2. Additionally, enhanced barrier signal and a modest increase in ventilation defects were also observed, primarily at week-2.

Figure 4(A,B) show the functional ventilation, barrier:gas, and RBC:gas binning maps for representative healthy rats. On the left, the distributions for each are shown along with the reference distribution (dotted line) from healthy animals. The panels on the right are the binning color maps, overlaid onto anatomical ^1^H MRI, with color bars showing the order of bins. Average values for the ventilation defect percentage (VDP), percentage of RBC defects (RBC_defect_%), and percentage of hyperintense barrier signal (barrier_high_%) are reported in Table 1.

The control animals showed homogeneous ventilation, largely devoid of defects. The barrier images exhibited a few baseline defects, mostly near the base of the lungs, as well as a few regions of moderately high uptake in the mid-lung region. The RBC images indicated signal that fell predominantly in the normal reference range, with the exception of defects below the heart, which are suspected to be from motion-associated artifact rather than having biological significance.

**Figure 5:**
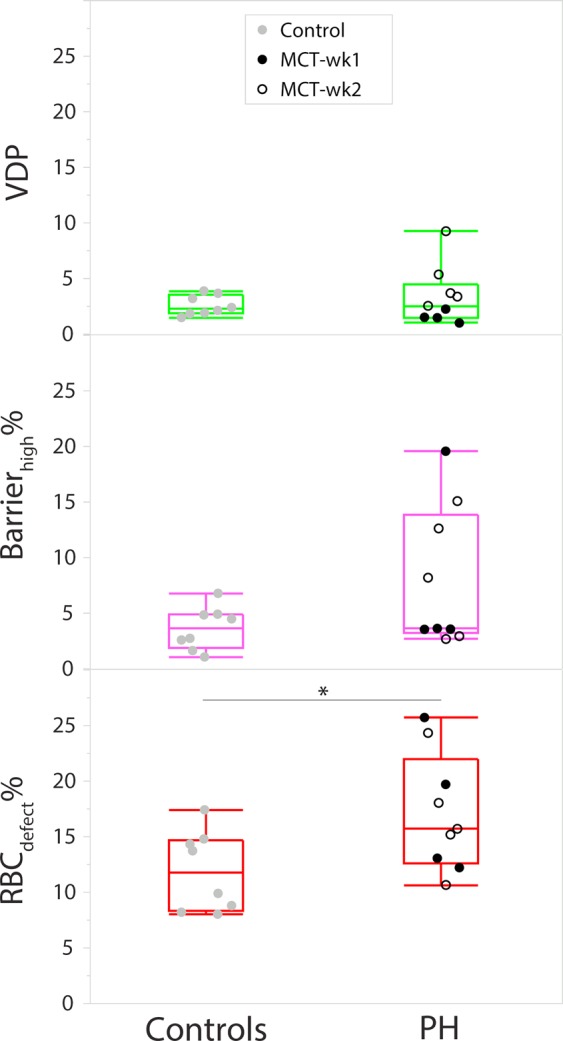
Box-and-whisker plots for VDP, barrier_high_%, and RBC_defect_% for the control and PH groups. Individual values for all rats are plotted as circles. Whiskers show minimum and maximum values.

Ventilation and gas-exchange maps in representative rats from the PH group are seen in Fig. 4C–F, and box-and-whisker plots comparing VDP, barrier_high_% and RBC_defect_% between the control and PH groups are seen in Fig. 5. The key features in these maps and differences from controls are discussed in the following sections for each ^129^Xe compartment.

### Ventilation

Ventilation was largely normal in the PH group (Fig. 4C,D,F); only a few animals exhibited defects and regions with low ventilation, which were observed only at week-2 and mostly confined to the periphery of the lungs (Fig. 4E.) Ventilation defects were not significantly different between the controls (VDP = 2.5 ± 0.9%) and entire PH group (VDP = 3.3 ± 2.6%), but were found to be significantly greater in the MCT-wk2 group (VDP = 4.7 ± 2.7%), compared to the controls (P = 0.048) and MCT-wk1 group (VDP = 1.5 ± 0.5%, P = 0.020) (Fig. 5). Additionally, regions with low ventilation (orange cluster) were also significantly greater in the MCT-wk2 group relative to the other two groups (P < 0.037) and covered a greater volume than ventilation defects (37.7 ± 20.4% in MCT-wk2; 15.1 ± 4.2% in MCT-wk1; 16.0 ± 1.9% in controls).

### Barrier:gas

Barrier_high_% was greater in the PH group (7.9 ± 6.3%) relative to controls (3.5 ± 1.9%), but this difference was not statistically significant because of the large variability in the PH group. Some animals in the PH group exhibited normal ^129^Xe uptake in barrier tissues (Fig. 4C), whereas 4/9 animals exhibited regions where it was considerably elevated (Fig. 5). In some animals, barrier_high_% was ~4× greater than controls. Moreover, like ventilation defects, high barrier-uptake was also predominant at week-2 (barrier_high_% = 8.3 ± 5.4%), and also observed mostly at the periphery of the lungs (Fig. 4D–F).

### RBC:gas

Defects in RBC-transfer were observed in the PH group at both week-1 and week-2. These defects were mostly confined to anterior lung regions. Such anterior RBC-defects are exhibited by all four PH rats in Fig. 4, and mostly existed in the absence of any abnormalities in ventilation or barrier-uptake, and vice versa. For example, Fig. 4C shows a large RBC-transfer defect in the left-lower lobe (red arrows), whereas ventilation and barrier-uptake appear normal in this region. Conversely, Fig. 4D exhibits high barrier signal in the central slice, but normal RBC-transfer.

RBC-transfer defects in the PH group were 17.1 ± 5.3% vs. 11.8 ± 3.6% in the control group (P = 0.034), with two PH rats showing significantly elevated defects comprising 25% of the lung (Fig. 5). The reduction in spectroscopic RBC:barrier at week-1 translated to an increased RBC_defect_%, although this change was not statistically significant. RBC_defect_% was also not significantly different between MCT-wk1 and MCT-wk2 rats.

## Discussion

### Reduced ^129^Xe transfer to RBCs

Both ^129^Xe spectroscopic RBC:barrier ratio and the RBC:gas color maps revealed significantly reduced ^129^Xe transfer to RBCs in the PH group. In this group, the spectroscopic RBC:barrier was strongly correlated with imaging-derived RBC_defect_% (*r* = −0.73, P = 0.027). However, in the control group, no correlation was observed. This shows that the RBC defects in healthy rats—observed primarily at the base of the heart—do not have physiological significance, but likely result from systematic artifacts. The most likely of these are susceptibility effects arising near the heart/lung border and motion of the heart and diaphragm. Such motion could disproportionately affect the dissolved-phase images, which were acquired without respiratory gating. This reasoning likely also applies to the barrier images, where defects are normally only observed in emphysematous regions^17^. Such systematic errors suggest imaging would benefit from acquisitions using faster read-outs and prospective gating.

The reduction of spectroscopic RBC:barrier in the PH group reflects reduced gas-exchange efficiency from a combination of diffusion limitation caused by increased interstitial barrier thickness, and from reduced perfusion. This metric has been shown clinically to correlate strongly with DL_CO_ (diffusive capacity of the lung for carbon monoxide) and has primarily been used to quantify diffusion limitation in diseases such as IPF^18^ and in their animal model analogs^19^. However, in a recent case report, the RBC:barrier ratio was reported for the first time to be reduced in PH and pulmonary veno-occlusive disease^13^. Although this was attributed to be dominated by perfusion limitation, this could not be confirmed at the time. However, it is known that a common finding in pulmonary vascular disease is a reduction in DL_CO_^20^, which is attributed to a combination of both lost capillary blood volume and worsening membrane diffusing capacity.

The ability to resolve the RBC-transfer and barrier-uptake into separate images provides a means to separately assess the effects of capillary blood volume and membrane diffusing capacity. In this study, quantitative mapping in all PH animals (Fig. 4) revealed reduced RBC-uptake to be mostly confined to the anterior lung and was generally not accompanied with any abnormality in barrier signal. This lends evidence to suggest that the RBC-transfer defects observed in these regions were not caused by diffusion limitation, but rather perfusion defects. Interestingly, these anterior regions represent the gravitationally dependent lung for rats, where the hemodynamic stress is highest, and thus vascular remodeling in response to injury is expected to be greatest^21,22^. Additionally, the presence of edema in posterior lung regions could lead to hyperventilation of the anterior lung, resulting in pressure-induced reduction of capillary blood volume that would further reduce RBC:gas signal. However, dissolved-phase imaging was done over the entire respiratory cycle, because of which, the bulk of the data was acquired during expiration. This should limit the degree to which inspiratory pressure would reduce RBC signal.

The reduction in RBC signal in the PH group was accompanied by an increase in chemical shift of the RBC resonance by 0.2 ppm (P = 0.039). This may be interpreted in light of previous studies in humans that have demonstrated the chemical shift of the RBC resonance to increase with blood oxygenation^23^. Therefore, the small but significant downfield shift observed in the PH group could reflect marginally higher blood oxygenation, caused by blood flowing more slowly through the gas-exchange region. However, this hypothesis should be tested in future *in vivo* and *in vitro* studies where blood oxygenation is explicitly controlled.

### Increased ^129^Xe uptake by barrier tissues

A striking finding in the PH group was the enhanced ^129^Xe uptake by barrier tissues in a subset of the animals. This enhancement was most notable at the periphery of the lung and gradually reduced to normal intensity toward the central lung. Such elevated barrier-uptake has been reported in patients with IPF^10^, where it follows a peripheral and basal pattern, and is thought to reflect regions of interstitial fibrotic thickening. In human IPF and rodent models of fibrosis^19^, high barrier signal is frequently accompanied by reduced gas-transfer to RBCs. Since the MCT model of PH is also known to demonstrate fibrosis^24^, it is plausible that it is this effect that is responsible for the observed high barrier-uptake. However, such a correlation could not definitively be identified in this cohort. It is therefore plausible that some high-barrier signal may have originated from pathology other than fibrosis. MCT is known to also induce interstitial and alveolar edema^25,26^. Uptake of ^129^Xe by interstitial fluid and that accumulated within alveoli could explain the enhanced barrier signal, while not restricting the diffusion of ^129^Xe into blood. This hypothesis is corroborated by the observation of edema on ^1^H MRI, most notably in the posterior lung. Since edema on ^1^H MRI and high barrier-uptake on ^129^Xe MRI were both mostly observed at week-2, this further suggests that these effects could be related. However, regions identified in ^1^H MRI as having edema were not ventilated, and therefore could not be assessed for gas-exchange abnormalities.

### Ventilation defects

Ventilation defects were modest in the cohort, and most prominent in MCT-wk2 rats. Like the high ^129^Xe barrier-uptake signal, the defects were mostly confined to the lung periphery. Ventilation defects were also accompanied by extensive regions of low ventilation. No direct spatial relationship between ventilation abnormalities and RBC-transfer defects was observed.

The late emergence of ventilation defects is suggestive that this could be related to late or more chronic stages of the model. The observation of ventilation defects is also consistent with the widespread pneumotoxicity of the MCT model^27^, which includes airway and alveolar dysfunction^28,29^. This is corroborated by the absence of ventilation in regions of extensive edema (Fig. 4E,F). Moreover, the peripheral location of defects is a feature also noted in human PAH^8,30,31^.

### Comparison of imaging and histology

Interestingly, the abnormalities observed with quantitative ^129^Xe MRI were somewhat disproportionate to the changes seen pathologically. Spectroscopic RBC:barrier correlated moderately with medial wall thickness (*r* = −0.57, P = 0.025). Notably, the correlation was absent with imaging metrics of ventilation, barrier signal, and RBC defects. Imaging revealed reduced gas-uptake by RBCs 1 week post MCT treatment, as well as regions of elevated barrier-uptake, suggestive of fibrosis, inflammatory processes, and edema. However, H&E histology showed significant medial wall thickening only at 2 weeks. This difference suggests that changes in gas-exchange physiology visualized with ^129^Xe MRI are more sensitive to the early manifestations of PAH, before it is obvious at a pathological level.

### Limitations

The monocrotaline model of PH was employed since it is technically straightforward, has been extensively studied, and is known to exhibit important aspects of human PAH such as pulmonary vascular remodeling and RV hypertrophy^14^. However, the effects of MCT are manifold, and the model can also exhibit pulmonary interstitial edema, myocarditis, hepatic veno-occlusive disease and renal alterations, which are not associated with human PAH^32^. Despite the imperfections of the model, it was encouraging that ^129^Xe MRI was able to identify several of its salient characteristics including edema and peripheral ventilation defects.

It was further notable at week-1, that abnormalities observed in imaging were found to be more substantial than those observed with histology. However, histological analysis was limited in that sections were only obtained from the right upper lobe, which may not have represented the magnitude of injury to the rest of the lung. For instance, the MCT-wk1 rat in Fig. 4C does not show any RBC-transfer defects in the right upper lobe. Furthermore, while histology, revealed significant injury at week-2, xenon transfer to RBCs did not further worsen between the week-1 and week-2 time points. We suspect that these discrepancies may be limited by the small sample sizes, and point to the need for future studies that are sufficiently powered to detect changes between these early time points, while also adding a more severe time point (3–4 weeks) to permit evaluating further progression of the primary imaging end point.

Regarding image acquisition, the dissolved-phase image was acquired without respiratory gating to maximize SNR, but at the cost of introducing some errors in gas-exchange mapping. These manifested as defects below the heart and near the diaphragm. These systematic errors undesirably skew reference values and must be reduced in the healthy population. They could potentially be limited by employing a faster dissolved-phase readout, retrospective-gating of dissolved-phase MRI, or alternatively by also acquiring gas-phase MRI without gating.

Lastly, the current image acquisition scheme effectively probed only a single time point on the ^129^Xe-RBC replenishment curve. More comprehensive modeling of gas-exchange is possible by acquiring spectra at different replenishment time points. Through this method quantitative metrics such as alveolar septal thickness, gas-exchange time constant and hematocrit can be obtained^33,34^. However, this approach currently sacrifices spatial resolution and thus obscures pathological markers of heterogeneous disease. Thus, we elected to trade off analysis of temporal dynamics for 3D isotropic spatial resolution needed to capture the complex structural and functional abnormalities in the MCT model of PH.

## Conclusion

We have presented the first application of quantitative 3D ^129^Xe gas-exchange MRI to the well-established MCT rat model of PH. This proof-of-concept study demonstrates the sensitivity of ^129^Xe spectroscopy and imaging to detect several salient characteristics of PAH, even before the injury fully manifested in histology. Particularly notable was a significant reduction in ^129^Xe transfer to RBCs, which was significantly correlated in imaging and spectroscopy, and the ability to observe early disease before other structural abnormalities manifested. This study has demonstrated that HP ^129^Xe MRI has strong potential to be used to non-invasively monitor the progression of disease in preclinical models of PH and potentially assess response to therapy. Future studies will focus on monitoring PH at later time points in the MCT model and testing alternative PH models such as SuHx, along with monitoring response to therapy.

## Methods

### Animal preparation

All animal experiments were approved by the Duke University Animal Care and Use Committee (IACUC) and were conducted in accordance with IACUC guidelines and regulations (Protocol #A037–17–02). The study involved two groups of male Sprague Dawley rats (Charles River, Wilmington, MA, USA). The control group (N = 8, weight = 178 ± 29 g) did not receive any treatment. The PH group (N = 9) received a subcutaneous injection of MCT (Sigma-Aldrich, St. Louis, MO, USA) diluted to 60 mg/kg in isotonic normal saline, and sterile filtered through a 0.2 µm filter prior to administration. Only male rats were used in this study as female rodents are known to exhibit a variable response to MCT^35,36^. The weight of the rats at the time of injection was 155 ± 8 g. The injected rats were imaged at two time points post-injection: 1 week (MCT-wk1; N = 4) and 2 weeks (MCT-wk2; N = 5). Immediately after imaging, the rats were euthanized, and their lungs extracted for histology.

Prior to MRI, the animals were induced with 2.5% isoflurane, followed by an intraperitoneal (IP) 50 mg/kg dose of pentobarbital (Nembutal, Lundbeck Inc., Deerfield, IL, USA). The rats were then intubated with a custom, tapered 12 G/14 G catheter, positioned on the animal cradle, and connected to a constant-volume ventilator^37^. The rats were normally ventilated with a 2-ml tidal volume comprising 25% O_2_ and 75% N_2_; during imaging, N_2_ was substituted by HP ^129^Xe. Xenon gas for imaging was prepared by polarizing isotopically enriched xenon (85% enriched, Linde AG, Munich, Germany) to ~20% using a commercial polarizer (Model 9810, Polarean Inc., Durham, NC, USA). The breathing rate was set to 60 breaths/min, and each cycle comprised of 250 ms inspiration, 250 ms breath-hold, and 500 ms exhalation. The rat was then connected to physiological monitoring sensors and the animal cradle was slid into the magnet bore such that the lungs of the rat were at isocenter. A pressure sensor continuously measured the animal’s airway pressure, a pulse oximeter clipped to a hind limb measured the heart rate and SPO_2_, and a rectal fiber optic probe reported temperature. Body temperature was maintained between 36–37 °C by circulating warm air through the bore. Supplemental doses of pentobarbital (30% of the initial dose, IP) were administered every ~45 minutes to maintain a stable heart rate.

### MR imaging and spectroscopy

Each imaging session included anatomical ^1^H MRI, ^129^Xe spectroscopy and ^129^Xe MRI of ventilation, barrier-tissue uptake, and RBC-transfer. ^1^H and ^129^Xe MRI were conducted on a Bruker 7 Tesla magnet (BioSpec 70/20 USR Avance III with 440 mT/m gradients, running ParaVision 6.0.1). ^1^H MRI used a quadrature transmit/receive coil (Bruker model 1 P T9562V3), and ^129^Xe MRI used a home-built transmit/receive linear birdcage coil. For accurate positioning of animals within the coils inside the bore, the animal bed was positioned inside a cantilevered plexiglass tube around which the ^129^Xe and ^1^H coils could be swapped without moving the animal.

MR imaging and spectroscopy parameters are listed in Table 2. Prior to ^129^Xe MRI, ^1^H localizer scans were obtained to center the lungs of the rat within the RF coil. ^1^H localizers were followed by respiratory-gated ^1^H MRI using a 3D ultra-short echo time (UTE3D) acquisition. Next, ^129^Xe spectroscopy calibration scans were done to establish the ^129^Xe gas- and dissolved-phase excitation frequency, RF transmit power, and optimal echo time (TE_90_) for separation of ^129^Xe dissolved-phase MRI into its barrier and RBC compartments using the 1-point Dixon technique^38^.

**Table 2:** Parameters for ^1^H MRI, ^129^Xe gas MRI, ^129^Xe dissolved MRI, and ^129^Xe spectroscopy.

Parameters	^1^H MRI	^129^Xe gas MRI	^129^Xe dissolved MRI	^129^Xe spectroscopy
RF pulse	block pulse	block pulse	3-lobe sinc	3-lobe sinc
RF duration (µs)	100	100	310	310
Excitation frequency (MHz)	300.3	83.06	gas+17.51 kHz	gas+17.51 kHz
TE (ms)	0.4	TE_90_	TE_90_	TE_90_
TR (ms)	10	10	15	15
Flip angle	5°	15°	20°	20°
Bandwidth (kHz)	100	10	100	100
NEX	1	1	7	200
Data points per ray	64	64	64	512
Matrix size^3^	128	128	128	—
Field of view (mm)	50	50	100	—
Breath-hold gating	yes	yes	no	no
Rays (total)	14328	3582	2073	—
Rays per breath-hold	20	20	—	—
Breath-holds (total)	717	180	218	3
Scan time	11 m 57 s	3 m	3 m 38 s	3 s

The spectroscopic calibration scans were followed by UTE3D ^129^Xe gas-phase MRI acquired with the same FOV (5 cm) and resolution (0.39 mm isotropic) as ^1^H MRI. ^129^Xe gas-phase MRI was respiratory-gated, acquiring 20 radial views during each inspiratory breath-hold, over a total of 180 breaths, and consumed ~400 ml HP ^129^Xe. Finally, UTE3D ^129^Xe dissolved-phase MRI was acquired using a selective 310-µs 3-lobe-sinc pulse that limited off-resonance ^129^Xe gas signal to ≤10% of total dissolved signal. This acquisition used the spectroscopically-determined TE_90_ (248 ± 5 μs) and a rapid readout of 0.6 ms to sample the short-lived dissolved-phase signal (T_2_* ~ 0.5 ms). To maximize SNR, this image was acquired without respiratory gating, with 7 averages, and a 2 × larger FOV of 10 cm. All UTE3D acquisitions were reconstructed^39^ onto a 128 × 128 × 128 matrix.

^129^Xe dissolved-phase MRI was immediately followed by a spectroscopy acquisition using parameters identical to imaging to obtain the RBC:barrier ratio under the same steady-state ^129^Xe replenishment condition. This ratio was used for quantitative image processing.

### Collection of lung samples and histology

After imaging was complete, the animal was sacrificed and lungs were extracted, inflated, and fixed as previously described^9^. Briefly, while still in the thoracic cavity, the lungs were flushed with phosphate buffered saline by stabbing the right ventricular free wall with a syringe and injecting toward the pulmonary artery. The lungs were then inflated to a pressure of 20 cmH_2_O with 10% buffered-neutral formalin, and held in place for 5 minutes. The trachea was then tied off and lungs dissected out of the thorax and fixed with 10% buffered-neutral formalin. Following fixation, the right-upper lobe of the lung was sliced and processed for hematoxylin-eosin (H&E) staining to assess remodeling of the arterioles.

To assess pulmonary vascular remodeling, H&E stained samples were examined for 20- to 80-µm muscular arteries. The external and internal media perimeters of at least 5 muscular arteries for each blinded sample were measured using ImageJ and external and internal media radii were calculated using *r* = *perimeter / 2π*. The medial wall thickness was expressed as *(external media radii – internal media radii) / external media radii* or as a ratio of medial area to cross-sectional area using *(total vascular area* − *lumen area) / total vascular area*^40^. Quantifications were performed by investigators blinded to the experimental groups.

### Processing of spectra and images

MR images and spectra were processed by adapting custom routines in MATLAB and Python that have been routinely employed at our center for analysis of clinical ^129^Xe MRI^10,17,41^. Complex ^129^Xe spectra were fit to a combination of three Lorentzian line shapes to quantify the primary *in vivo* ^129^Xe resonances: gas-phase, barrier tissue, and RBCs. For each resonance, the amplitude, chemical shift, line width, and phase were determined. The ratio of RBC and barrier signal amplitudes (RBC:barrier) was calculated to quantify ^129^Xe transfer to RBCs.

The dissolved-phase image was separated into barrier and RBC images by applying a global phase shift to the real and imaginary components of the complex dissolved-phase image until the ratio of their image intensities matched the spectroscopically determined RBC:barrier^17^. ^129^Xe gas- and dissolved-phase images were then processed to create gas-exchange maps. Briefly, the ^1^H image was first segmented to obtain a lung mask that confined analysis of ^129^Xe images to the thoracic cavity. A thoracic cavity mask was employed based on prior knowledge that the MR acquisition protocol (20° flip angle, 15-ms TR) ensured that the dissolved-phase ^129^Xe signal is confined to arise only from the pulmonary gas-exchange regions of the lung, and not from downstream regions (major vessels, heart). This confinement is best understood through the framework of Ruppert *et al*., where a combination of 20° flip angles applied at a TR of 15 ms yields an effective RF-induced T_1_ of 0.25 s^42^. Because this is short compared to the capillary transit time in rats of ~0.51 s^43^, ^129^Xe magnetization does not reach distal organs.

The ^1^H image and its mask were then registered to the ^129^Xe ventilation image. The mask was further refined by removing conducting airways (segmented from the ^129^Xe ventilation image) and regions with ventilation defects, to eliminate regions in the lung that do not participate in gas-exchange. Next, the ventilation, barrier, and RBC images were corrected for differences in flip angle and T_2_* decay, after which the barrier and RBC images were normalized by dividing by the ventilation image on a pixel-by-pixel basis to generate quantitative maps of ^129^Xe barrier-uptake and ^129^Xe RBC-transfer (referred to as barrier:gas and RBC:gas, respectively). The ventilation images were also normalized by their top percentile values in order to compensate for the tail of high intensity values in the histogram and re-scale the intensities to a range of 0 to 1^44^. This percentile-based rescaling approach eliminates subjective normalization with a single high intensity threshold, and has been demonstrated to generate normal reference histograms that enable robust ventilation mapping of clinical ^129^Xe images.

**Figure 6:**
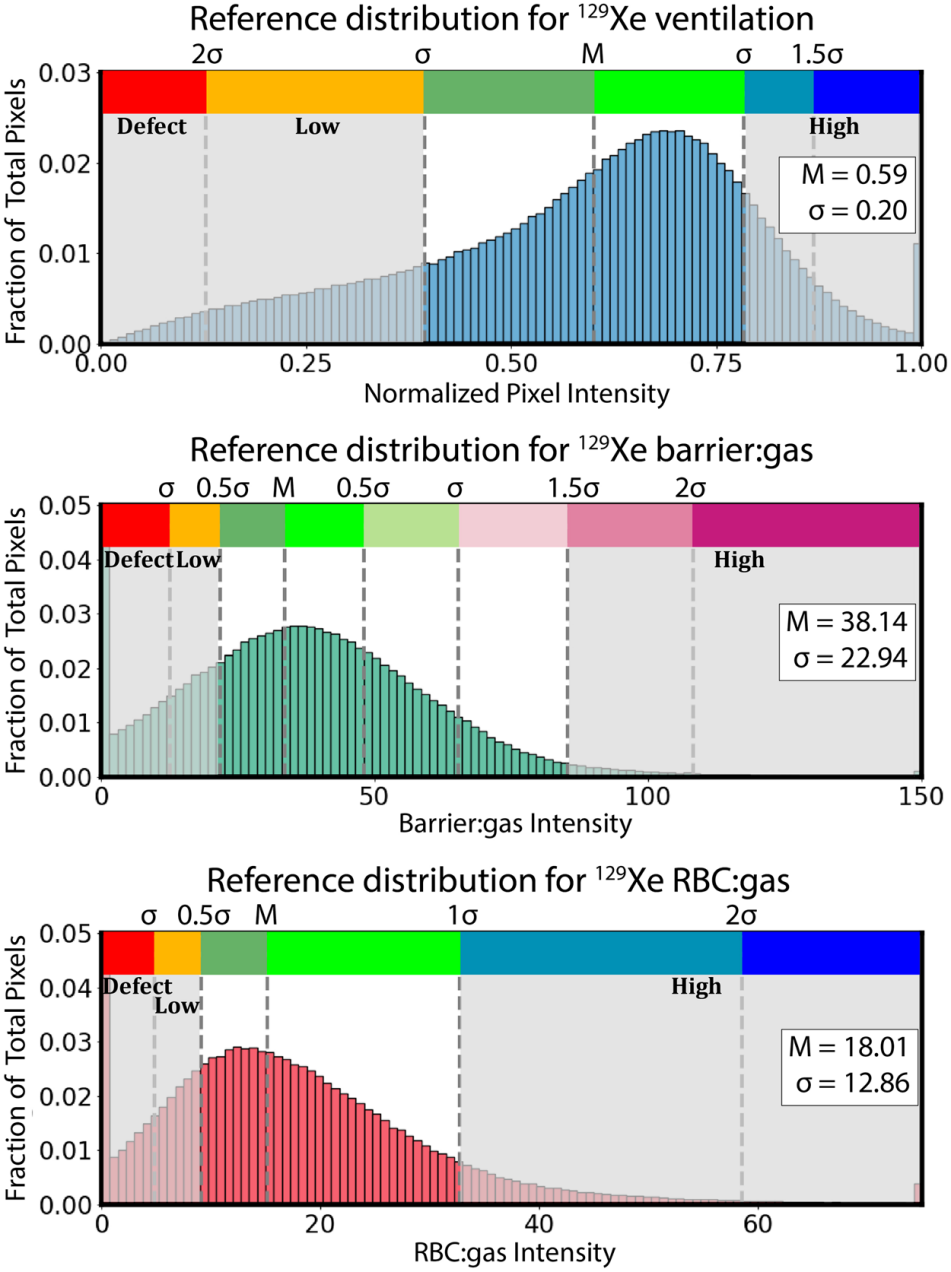
Reference distributions for ^129^Xe ventilation, barrier:gas, and RBC:gas images. The distributions underwent a Box-Cox transform, and binning thresholds were determined from the mean and multiples of standard deviation of the transformed distributions. The thresholds are indicated above the distributions. Shaded areas indicate bins corresponding to defects, low signal intensity, and high signal intensity.

Finally, ^129^Xe ventilation, barrier:gas, and RBC:gas images underwent binning analysis. This employed the aggregate histograms for all the three ^129^Xe images (ventilation, barrier:gas, and RBC:gas) generated from the healthy rat population. Because of the non-Gaussian nature of the reference histograms, binning thresholds could not directly be established based on the mean and standard deviation of the distribution. To accommodate the non-Gaussian reference histograms, a one-parameter Box-Cox transform was applied to render them approximately Gaussian^45^. In this transformed domain, the mean and standard deviation were calculated to determine the threshold values. These values were then transformed back to the original non-Gaussian distribution to create uneven binning thresholds. Figure 6 shows the reference distributions for all three ^129^Xe compartments, along with the positions of the thresholds and colors of the bins. The ventilation and RBC:gas distributions used 6 bins: the first bin (red) shows regions of signal void or defects, the second bin (orange) shows low intensity regions, the next two bins (green) show intensities close to the mean of the distribution, and the final two bins (blue) show regions of high intensity signal. The barrier:gas distribution used 8 bins to capture its broader dynamic range, with the highest 3 bins using shades of purple to highlight regions of elevated barrier signal, a hallmark of interstitial thickening^10^. Our binning analysis for rats resulted in a color display that was consistent with our standard analysis of clinical images^17^.

### Statistical analysis

Statistical analysis was performed using JMP Pro 13.1 (SAS Institute Inc., Cary, NC, USA). Given the small sample size, non-parametric tests were used. Five imaging-based metrics of lung function were compared between the control and PH groups using the Mann-Whitney *U* test: the chemical shifts of the RBC and barrier resonances, spectroscopic RBC:barrier, ventilation defect percentage (VDP), percentage of RBC defects (RBC_defect_%), and percentage of hyperintense barrier signal (barrier_high_%). These metrics were selected because of their relevance in identifying ventilation and gas-exchange abnormalities in various lung diseases in humans^10,11,13^. Histology samples were analyzed for medial wall thickness and compared between groups.

Next, the PH group was split into MCT-wk1 and MCT-wk2, and these two groups along with the control group were tested for differences in the same 5 metrics using the Kruskal-Wallis test, which if significant, was followed by post-hoc comparisons using Mann-Whitney *U* tests. The first test—with all the PH rats pooled together—was strengthened by a larger sample size and would also represent a realistic scenario analogous to a clinical study where patients within a disease group present with different levels of disease progression. The second test—with the PH rats separated by time point—was done to identify trends in the progression of the disease, at the cost of being underpowered and susceptible to outliers. Lastly, the spectroscopically determined RBC:barrier was correlated with the RBC_defect_% using linear regression analysis and the Pearson correlation coefficient (*r*). Imaging-based metrics were also correlated with medial wall thickness.

All tests were considered significant if the P-value was <0.05. The P-value was not corrected for multiple comparisons given the small sample size, and to preserve sensitivity to small differences by avoiding possible type-II errors caused by a conservative significance level.

## Data Availability

The datasets generated during and/or analyzed during the current study are available from the corresponding author on reasonable request.
